# Relationship between pyroptosis-mediated inflammation and the pathogenesis of prostate disease

**DOI:** 10.3389/fmed.2023.1084129

**Published:** 2023-01-19

**Authors:** Ming Zhao, Jun Guo, Qing-He Gao, Hao Wang, Fu Wang, Zi-Rui Wang, Sheng-Jing Liu, Ying-Jun Deng, Zi-Wei Zhao, Yue-Yang Zhang, Wen-Xiao Yu

**Affiliations:** ^1^Graduate School, Beijing University of Chinese Medicine, Beijing, China; ^2^Department of Andrology, Xiyuan Hospital of China Academy of Chinese Medical Sciences, Beijing, China; ^3^Department of Andrology, Wangjing Hospital of China Academy of Chinese Medical Sciences, Beijing, China

**Keywords:** prostate disease, pyroptosis, inflammasome, cell death, prostate cancer

## Abstract

The largest solid organ of the male genitalia, the prostate gland, is comprised of a variety of cells such as prostate epithelial cells, smooth muscle cells, fibroblasts, and endothelial cells. Prostate diseases, especially prostate cancer and prostatitis, are often accompanied by acute/chronic inflammatory responses or even cell death. Pyroptosis, a cell death distinct from necrosis and apoptosis, which mediate inflammation may be closely associated with the development of prostate disease. Pyroptosis is characterized by inflammasome activation via pattern recognition receptors (PRR) upon recognition of external stimuli, which is manifested downstream by translocation of gasdermin (GSDM) protein to the membrane to form pores and release of inflammatory factors interleukin (IL)-1β and IL-18, a process that is Caspase-dependent. Over the past number of years, many studies have investigated the role of inflammation in prostate disease and have suggested that pyroptosis may be an important driver. Understanding the precise mechanism is of major consequence for the development of targeted therapeutic strategies. This review summarizes the molecular mechanisms, regulation, and cellular effects of pyroptosis briefly and then discuss the current pyroptosis studies in prostate disease research and the inspiration for us.

## Highlights

- Pyroptosis, a cell death distinct from necrosis and apoptosis, which mediates inflammation may be closely associated with the development of prostate disease.
- More and more studies have shown that pyroptosis may play an important role in prostate diseases, such as prostate cancer, benign prostatic hyperplasia, and prostatitis.
- Understanding the role of pyroptosis in prostate disease has important guiding significance for the design of treatment strategies.

## 1. Introduction

The prostate is the largest unpaired substantial organ of the male genital appendages, consisting of glandular tissue, smooth muscle, and connective tissue. The prostatic portion of the urethra is threaded through its substance. Its secretions are the major component of semen and have a nutritional and sperm-activating effect. When the tissue structure of the prostate gland becomes abnormal, it is referred to as a prostate disorder, which means that diseases such as prostate cancer, benign prostatic hyperplasia (BPH), and prostatitis may have occurred. However, maintaining the normal structure and function of the prostate requires a balance between cell formation and death in the prostate tissue (including prostate epithelial cells, smooth muscle cells, fibroblasts, and endothelial cells) (1).

Under physiological conditions, cells in an organism live in an orderly cellular society according to definite rules. Cell death starts when cells are subjected to some strong external stimulus or when their own mechanisms develop some kind of irreparable problem. Common forms of cell death include necrosis, apoptosis, and pyroptosis (2). Upon stimulation of cells, perforation of the plasma membrane is mediated by GSDM family proteins and accompanied by the release of inflammatory molecules, that is pyroptosis, a procedure distinct from apoptosis or necrosis (3). Morphologically, the pyroptosis cells showed agglutination of nuclei, positive *in situ* deoxyribonucleic acid (DNA) fragment labeling, and positive membrane association protein staining. The cytoskeleton proteins are degraded, and the membranes show 1-2 nm oligomeric protein pores and progressive dissolution of the plasma membrane. Its intracellular pro-inflammatory small molecules are effluxed and cause a strong inflammatory response (4). Notably, compared to necrosis, the plasma membrane of pyroptosis cells gradually dissolves and the contents are released more slowly (3). When apoptosis occurs, the DNA is fragmented and the plasma membrane remains intact without leakage of contents (5). Given the unique mechanism of pyroptosis, its role in prostate disease has been gaining attention. This review provides a current overview of the evidence and functional role of pyroptosis in prostate disease and discusses the molecular pathways involved in the treatment of prostate disease.

## 2. Overview of pyroptosis

**FIGURE 1:**
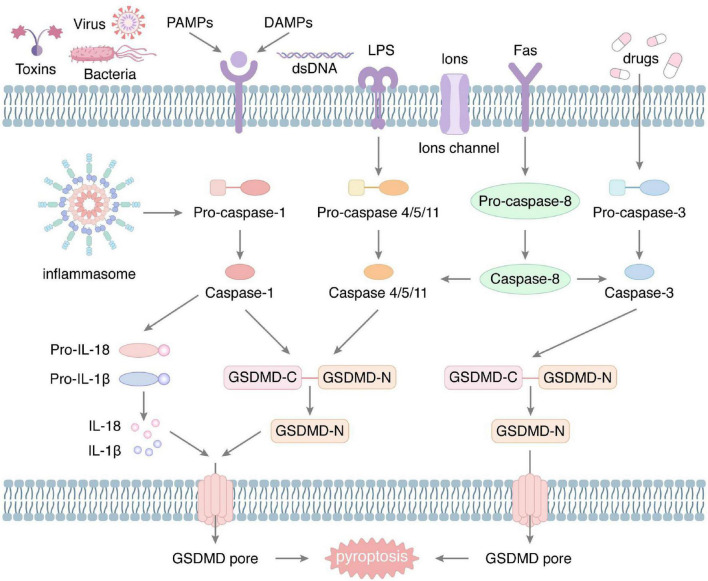
The molecular mechanism of pyroptosis. The classical pyroptosis is produced under the stimulation of a variety of external factors, such as bacteria, viruses, and toxins, which can activate the inflammasome. Activated inflammasome induces cleavage of pro-caspase-1 to form Caspase-1. Subsequently, Caspase-1 cleaves GSDMD to produce the N-fragment of GSDMD, which aggregates to generate plasma membrane pore, resulting in rupture of the cell membrane. Also, Caspase-1 promotes the maturation and secretion of IL-1β and IL-18. In addition, external stimuli can activate Caspase-4/5/11, Caspase-3, and Caspase-8, mediating the non-classical pyroptosis. They all eventually lead to perforation of the plasma membrane.

In 1992, Zychlinsky et al. first observed the phenomenon of pyroptosis in macrophages, which was caused by infection with *Shigella flexneri* (6). In 2001, Cookson et al. coined the name of pyroptosis (7). In recent years, with increasing research, it has been confirmed that both pathogenic infection and endogenous damage can induce pyroptosis. Depending on the Caspases, pyroptosis can be divided into classical and non-classical pathways, with the commonality that both perforate the cell membrane by regulating the cleavage of gasdermin-D (GSDMD) at specific sites (Figure 1).

### 2.1. Molecular mechanisms about the classical pyroptosis

#### 2.1.1. Caspases-1 mediate the classical pyroptosis

Caspase-1 exists in the cytoplasm as an inactive zymogen with a relative molecular mass of about 47,000 daltons and is a human-mouse shared cell death-associated protein. Upon stimulation by pathogen-associated molecular patterns (PAMPs)/damage-associated molecular patterns (DAMPs), the intracytoplasmic PRR is activated to initiate an oligomerization reaction that selectively binds pro-caspase-1 to apoptosis-associated particulate proteins. At this point, pro-caspase-1 hydrolyze and releases p20 and p10 subunits, which polymerize into biologically active tetramers, resulting in the formation of specific inflammatory complexes, namely inflammasome (8). The aspartate residues on GSDMD are cleaved by Caspase-1 to form active amino N terminus and carboxyl C terminus, of which the lipid-selective N terminus can specifically bind lipid components on the cell membrane and oligomerize to form a ring-like hollow structure with a diameter of 10–21 nm (9). This structure disrupts the osmotic pressure balance inside and outside the membrane, and the cell then lyses and dies. Meanwhile, Caspase-1 cleaves pro-IL-1β and pro-IL-18 to form mature IL-1β and IL-18. After cell membrane perforation, IL-1β and IL-18 are secreted extracellularly via pore proteins to induce inflammatory responses, activate the pyroptosis pathway of other cells, produce chemotactic effects, and cause other immune cells to target chemotaxis and amplify inflammatory effects (10).

#### 2.1.2. Inflammasome associated with the classical pyroptosis

Inflammasomes are intracellular complexes involved in intrinsic immunity and usually consist of three parts: receptor proteins, adapter proteins, and effector proteins, which differ in their final structure depending on the protein substrate. Upon recognition of a specific PAMP or DAMP, the intracytoplasmic PRR activates and initiates the inflammasome assembly process, recruiting the adapter apoptosis-associated speck-like protein containing (ASC). Generally, ASC contains two structural domains, pyridine (PYD) and caspase recruitment domain (CARD), while some inflammasomes do not possess PYD (11). ASC activates Caspases via CARD, which further activates the inflammatory response downstream. To distinguish different inflammasomes, they can be classified into nucleotide-binding oligomerization domain (NOD) like receptor (NLR) family and absent in melanoma-2 like receptor (ALR) family, etc. according to the receptor proteins (12).

##### 2.1.2.1. NLR family

NLR family, including nucleotide-binding domain leucine-rich repeats protein (NLRP)1, NLRP3, NAIP-NLRC4, NLRP6 and NLRP9b, which all initiate the pathway of pyroptosis (13). NLRP1 is the earliest identified inflammatory vesicle and is widely present in human and murine macrophages, differing structurally in that murine NLRP1 has three homologous structures (NLRP1a-1c) and generally lacks PYD (14). NLRP1 specifically recognizes *Bacillus anthracis*, *Shigella flexneri*, fungi, *Toxoplasma gondii*, and Val-boroPro, a non-selective inhibitor of post-proline-cleaving serine proteases, and activates Caspase-1 to initiate the pyroptosis (14–16). The NAIP structure of the NAIP-NLRC4 inflammasome recognizes a variety of PAMPs, including needle-like proteins, flagellin, and rod-shaped proteins of *Toxoplasma gondii* (17, 18). Upon recognition of specific PAMPs, intracellular NLRC4 is activated and recruits ASC, which activates Caspase-1 and initiates the classical pathway of pyroptosis. In addition, NLRP6 and NLRP9b are equally capable of recognizing specific patterns to initiate classical pyroptosis, but they are mainly expressed in intestinal epithelial cells (19, 20).

##### 2.1.2.2. AIM2 inflammasome

Absent in melanoma-2 (AIM2) inflammasome is mainly composed of the N-terminal PYD domain and C-terminal HIN domain. It forms an AIM2 oligomer after binding to double-stranded DNA. AIM2 inflammasome, as a DNA sensor of innate immunity, can specifically identify and detect mutated or misplaced DNA molecules. AIM2 can not only directly detect DNA damage within the nucleus (21), but also recognize foreign cytoplasmic DNA (22). AIM2 aggregates the ligand ASC under PYD PYD interaction, which leads to the formation of the fibrous superstructure of ASC protein, and further occurs the aggregation of Caspase-1 and shear ripening of IL-1β (23). AIM2 inflammasome can be activated by damaged double-stranded DNA, causing the release of inflammatory factors and GSDMD mediated pyroptosis (24). While cytoplasmic dsDNA/AIM2-associated pyroptosis leads to macrophage dysfunction (25). Although pyroptosis is an important link in many diseases, the AIM2 inflammasome as a trigger of pyroptosis has not attracted enough attention.

##### 2.1.2.3. Pyrin inflammasome

Pyrin is an atypical NLR protein encoded by the *MEFV* gene, consisting of a PYD, two B-boxes, and a coiled-coil structural domain (26). Pyrin binds ASC, a ligand-protein for inflammasome (27), and Pyrin overexpression forms an ASC-dependent, caspase-1-activating complex (28), suggesting that Pyrin may form an inflammasome. The current study shows that the inactivation of RhoA leads to dephosphorylation of Pyrin, releasing the bound 14-3-3 protein, which activates the downstream inflammasome pathway and promotes the release of IL-1β (29). Activation of Pyrin inflammasome provides a substrate ready to mediate pyroptosis, but the precise mechanism remains unclear.

### 2.2. Molecular mechanisms about the non-classical pyroptosis

#### 2.2.1. Caspases-4/5 and Caspases-11 mediate the non-classical pyroptosis

Human-derived Caspases-4/5 and murine-derived Caspases-11 are homologous isomers with highly similar protein structures and essentially the same physiological functions. Unlike Caspases-1, in addition to specific inflammasome, pro-caspases-4/5 and pro-caspases-11 are able to directly recognize lipopolysaccharide (LPS), oxidized-1-palmitoyl-2-arachidonyl-sn-glycerol-3-phosphocholine (Ox-PAPC) signaling, which activates and induces pyroptosis. Upon stimulation by inflammasome, Caspases-11 activates and specifically binds intracellular lipoproteins, acting on GSDMD to release the N-terminal and C-terminal ends, causing perforation of the cell membrane (30). Meanwhile, activated Caspases-11 activates Pannexin 1 (Panx 1) channels in the membrane, induces adenosine-triphosphate (ATP) secretion from the cells, and activates purinergic ligand-gated ion channel 7 (P2 × 7) molecules, which activate downstream pro-caspases-1, thus initiating the classical pyroptosis and releasing IL-1β and IL-18.

#### 2.2.2. Other caspase family members and pyroptosis

Nowadays, Caspase-3 and Caspase-8 have also been shown to be closely associated with pyroptosis. In general, Caspase-3 is usually produced by the apoptotic pathway initiated by mitochondrial inducible factors and is considered to be an important link in apoptosis. Caspase-3 can induce a pyroptosis-like process in tumor cells that are otherwise in the process of apoptosis via gasdermin-E (GSDME), a homologous protein of GSDMD (31). Although Caspase-3 is able to mediate the pyroptosis-like process via GSDME, cell death in this process is atypical compared to pyroptosis.

Caspase-8 is the initiator of exogenous apoptosis and inhibits receptor-interacting protein kinase 3 (RIPK3) and mixed lineage kinase domain-like protein (MLKL)-mediated necrosis. Some recent evidence suggests that Caspase-8 can cleave GSDMD and thus mediate pyroptosis (32–34). In addition, Caspase-8 can promote pyroptosis by acting as a scaffolding protein that drives inflammatory factor production and activates Caspase-1 (35). Of note, the involvement of Caspase-8 in pyroptosis occurs simultaneously with apoptosis, and together they inhibit necrosis and reduce cell damage.

### 2.3. NLRP3 inflammasome

NLRP3 inflammasome is a multiprotein complex that comprises the nod-like receptor protein NLRP3, the adapter ASC, and the effector pro-caspase-1. The NLRP3 is connected by three homologous domains, which are leucine-rich repeat at the C-terminal, nucleoside triphosphatase domain at the center, and pyrin domain at the N-terminal. Intracellular NLRP3 content is extremely low at the tranquillization status. Once activated, the NLRP3 inflammasomes act as platforms to trigger Caspase-1, facilitate cytokine release, and induce pyroptosis (36).

**FIGURE 2:**
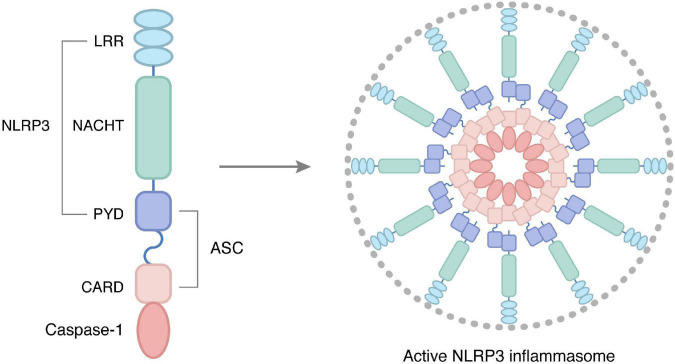
Structure and activation of NLRP3 inflammasome, as core proteins in the NLRs family, are important members of the body’s intrinsic immune system, and their abnormal activation is closely related to various chronic inflammatory conditions. NLRP3 inflammasome consists of three parts: receptor, adapter, and effector, namely NOD-like receptor protein 3, junction molecule apoptosis-associated protein ASC, and cysteine aspartate protease. In particular, the receptor NLRP3 is articulated by three homologous structural domains: the C-terminal leucine-rich repeat (LRR), the central nucleoside triphosphatase domain (NACTH), and the N-terminal pyrindomain (PYD). In the resting state, NLRP3 expression in cells is at very low levels, and upon binding to signals mediating NLRP3 inflammasome initiation, activates NLRP3 inflammasome thereby participating in the inflammatory response.

The classical NLRP3 inflammasome working procedure is completed by priming and activation (37), which are two parallel and independent steps (Figure 2). The transcription factor nuclear factor-kappa B (NF-κB) plays a vital role in priming the NLRP3 inflammasome. Under priming stimuli, some receptors such as toll-like receptors (TLRs), NLRs, or cytokine receptors activate NF-κB, which upregulates the expression of NLRP3 and pro-IL-1β (38, 39). Interestingly, the priming stimuli did not affect the expression of ASC, pro-caspase-1, and pro-IL-18 (38). Thus, it provides the required protein for the activation of the NLRP3 inflammasome.

There are mainly four patterns have been observed in activating the NLRP3 inflammasome. The first model holds that bacterial toxins and particulate matter trigger the formation of membrane pores that accelerate K^+^ efflux, which induces the assembly and activation of the NLRP3 inflammasome (40). The second model is that NLRP3 agonists are engulfed by cells, resulting in lysosomal rupture, releasing cysteine cathepsins, and inducing NLRP3 inflammasome assembly and activation (41). The third mode is that a range of situations of host ‘danger’, such as infection and metabolic dysregulation followed by reactive oxygen species (ROS)-generating mitochondria can activate the NLRP3 inflammasome (42). Besides, the calcium-sensing receptor (CASR) also plays an irreplaceable role in activating the NLRP3 inflammasome via increasing intracellular Ca (2+) and decreasing cellular cyclic AMP (cAMP) (43). In addition to the above four modes, the role of chloride ion efflux (44), metabolic changes (45), and trans-Golgi disassembly in the activation of NLRP3 inflammasome cannot be ignored. At present, our understanding of the NLRP3 inflammasome activation pathway is not systematic and comprehensive, and there are still many models to be found.

## 3. Pyroptosis in prostate disease

### 3.1. Prostate cancer

Prostate cancer (PCa) is a malignant tumor that occurs in the epithelium of the prostate gland and is one of the most common malignant tumors of the male reproductive system. The International Agency for Research on Cancer reported that the new cases and deaths of PCa worldwide in 2020 were 1.414 million and 0.375 million, respectively (46). This undoubtedly places a heavy burden on individuals, families and society. Therefore, there is an urgent need to conduct in-depth studies on the pathogenesis of PCa in order to provide effective therapeutic strategies for its targeted treatment. Current evidence suggests that pyroptosis may provide new insights into the understanding of PCa pathogenesis. Hu et al. analyzed the prognostic value of pyroptosis-related genes in PCa patients using biomedical databases and identified several pyroptosis-related genes that can predict the prognosis of PCa (47). The tumor microenvironment (TME) has been shown to play a key role in tumorigenesis, immune progression, escape and metastasis. Wang et al. further validated the relationship between the pyroptosis subtype and TME of PCa (48). Pyroptosis, as an inflammatory programmed cell death, is involved in the host protective immunity (49), which may lead to alterations of the immune microenvironment. A comprehensive and in-depth understanding of the TME by pyroptosis in PCa is needed. Zhang et al. identified several pyroptosis-related genes that may impact the prognosis of PCa patients by altering the TME (50), but the precise mechanism has yet to be uncovered. These findings indicate that pyroptosis is closely related to the development of PCa.

Abnormal alterations in various signaling pathways that regulate proliferation, migration, and apoptosis play key roles in tumor development. The main pathways that have been identified in studies of pyroptosis in PCa include Caspase-1, Caspase-4/5/11 and Caspase-3 pathways. The Caspase-1 pathway is a classical signaling pathway that is activated by inflammasome signaling and promotes pyroptosis in PCa. PCa and paraneoplastic tissues are accompanied by a large inflammatory response and inflammatory cell infiltration (51), and the inflammatory TME is likely important in promoting tumor cell growth. Cytokines released by inflammatory cells can promote tumor growth. The inflammatory response also activates transforming growth factor-β (TGF-β), thereby inhibiting Caspase-1 expression and IL-1β maturation and release (52). This allows tumor cells to be spared from pyroptosis and continue to grow. NLRP12, an inflammasome associated with pyroptosis, promotes the development of PCa by regulating Caspase-1 and downstream IL-1β and IL-18 (53). NF-κB and signal transducers and activators of transcription 1 (STAT1) can also induce pyroptosis by regulating Caspase-1 or GSDMD protein expression, accompanied by a large release of inflammatory factors, which acts as a tumor growth suppressor (54). LPS are involved in the proliferation, invasion and metastasis of PCa cells. Intracellular LPS activates the Caspase-4/5/11 pathway and induces pyroptosis, which in turn inhibits tumor growth. In PCa cells, alterations in the endoplasmic reticulum structure promote LPS synthesis, leading to changes in Caspase-4 expression, which can induce programmed cell death (55). A retrospective study found that Caspase-5 mRNA expression was significantly higher in PCa patients, suggesting that the involvement of Caspase-5 in pyroptosis may be associated with the risk of PCa development (56). Caspase-3 expression was significantly decreased in PCa tissues (57). Plasmacytoma variant translocation 1 (PVT1), a long non-coding RNA, is also involved in the development of PCa, and knockdown of PVT1 resulted in significantly upregulated Caspase-3 expression in mouse PCa tissues. Chemotherapeutic drugs can exert anti-tumor effects by cleaving GSDME in PCa tissues through Caspase-3, which triggers pyroptosis (5). A recent study also showed that C10, a novel 3′,5′-diprenylated chalcone, activates Caspase-3 by inducing the protein kinase Cδ (PKCd)/c-Jun N-termital kinase (JNK) pathway, which in turn triggers cleavage of GSDME to execute pyroptosis in PCa cells (58). However, the mechanism of Caspase-3-mediated pyroptosis in PCa needs to be further elucidated.

### 3.2. Prostatitis

Prostatitis is one of the most common and confusing diseases in Urology and Andrology. Although prostatitis is not a direct life-threatening disease, it seriously affects the quality of life of patients. At the same time, its large patient population and high medical costs place a huge economic burden on public health (59). Prostatitis is an inflammatory disease that is linked to both infectious and non-infectious factors. Pyroptosis has turned out to be present in a variety of infectious and sterile diseases (60–64). The pyroptotic cells are able to promote the development of an inflammatory response by secreting the necessary cellular components and inflammatory mediators. Thus, pyroptosis may be a key contributor to cell death during prostatitis.

Activation of the NLRP3 inflammasome is the leading inducer of pyroptosis-mediated cell death. Lu et al. used a hormone-imbalance-induced chronic non-bacterial prostatitis (CNP) rat model and found that NLRP3 inflammasome activation triggers a series of inflammatory responses in the prostate glands of CNP rats. Interestingly, these effects may be attenuated by rapamycin-induced autophagy (65). Chen and colleagues showed that the NLRP3 inflammasome may regulate CNP cell autophagy by modulating the IL-6/STAT3 pathway (66). Whether the NLRP3 inflammasome causes pyroptosis remains to be further determined. While in the experimental autoimmune prostatitis (EAP) mouse model, apart from the attenuating effect of the NLRP3 inhibitor MCC950 (67), melatonin also inhibited the NLRP3 inflammasome signaling pathway by activating Sirt1, which reduced prostate inflammation and pelvic pain (68). Zang et al. found elevated expression of NLRP3, Caspase-1 and ASC in CNP rats, and Qianliexin capsule (QLX) suppressed the high expression of these proteins (69). Another study showed that the levels of Caspase-1 and Pyrin domain containing proteins 1 and 3 (NALP1 and NALP3) were increased in local carrageenan-induced prostate inflammation (70). Wang et al. detected high expression of heat shock protein 70 (HSP70) in thulium laser resected prostate (TmLRP) tissue in a beagle model. The expressions of ROS, NLRP3, Caspase-1 and IL-18 were significantly increased in human myeloid leukemia mononuclear cells (THP-1) under the stimulation of HSP70 and were reduced by the ROS inhibitor N-acetyl-l-cysteine (NAC). The expressions of IL-1β and IL-18 were inhibited by NLRP3 or Caspase-1 inhibitors. This suggests that activation of the ROS-NLRP3 signaling pathway induces sterile inflammation in the post-prostatectomy wound (71). This seems to be suggesting that this process may also occur in the prostate tissue. However, these studies only showed that the drivers of pyroptosis-mediated cell death were expressed in the prostate glands and mediate the inflammatory response. Further experimental detection of cell morphological changes and associated pathways is required to demonstrate the onset of pyroptosis.

*In vitro* stimulation of a prostate epithelial cell line (RWPE-1) by *Trichomonas vaginalis* induced the expressions of NLRP3, ASC, Caspase-1, and IL-1β, while silencing of NLRP3 and Caspase-1 attenuated *T. vaginalis*-induced IL-1β secretion (72). The study demonstrated that NLRP3 inflammasome activation occurs in RWPE-1 cells and promotes the release of inflammatory factors in PAMPs-induced prostatitis. Several studies have reported that *Propionibacterium acnes*, a slow-growing Gram-positive anaerobic bacillus is frequently detected in prostate tissue and is associated with acute and chronic prostate inflammation (73–75). Sahdo and collaborators found that Caspase-1 was abundantly expressed in neutrophils under stimulation of *P. acnes*, whereas only a moderate activation was seen in monocytes (76). These studies provide some evidence that the development of prostatitis may be associated with pyroptosis. Which cells undergo a pyroptosis program during prostatitis and the regulatory relationships between cells remain to be elucidated, however.

### 3.3. Benign prostatic hyperplasia

Benign prostatic hyperplasia (BPH) is a pathological change in which the prostate stroma and/or glands are enlarged (77). BPH is the most common cause of voiding disorders in middle-aged and older men, manifested by histological hyperplasia of the interstitial and glandular components of the prostate, anatomical enlargement of the prostate, clinical symptoms dominated by lower urinary tract symptoms, and urodynamic obstruction of the bladder outlet (78). The exact molecular mechanism of BPH is not well understood, and both excessive cell proliferation and restricted programmed cell death processes may cause an increase in cell numbers.

Although there is little direct evidence of pyroptosis in BPH, pyroptosis-mediated inflammation may play a role in this process. The role of activation of the inflammasome as the initial step of pyroptosis in BPH cannot be ignored. Inflammasome expression, such as AIM2, was significantly elevated in BPH tissue compared with normal prostate tissue (79). Activation of inflammasome assembly leads to the production and secretion of the pro-inflammatory cytokines IL-1β and IL-18, thereby perpetuating the inflammatory state associated with BPH (80, 81). Prostate epithelial cells that die from pyroptosis release pro-inflammatory stimuli, which can induce chronic inflammation and BPH. Subsequently, epithelial cells and surrounding stromal cells in BPH tissue undergo compensatory growth in response to the inflammatory stimulus (82, 83). Jiang and colleagues found that peroxiredoxin 3 (PRDX3) was highly expressed in prostatic epithelial cells of BPH patients and BPH-representative cell lines (84). Upon reduction of PRDX3 expression in BPH-1 cells, Caspase-1 and cleaved Caspase-1 expression were subsequently increased and decreased, respectively, indicating impaired activation of Caspase-1. Lactate dehydrogenase (LDH) is released from pyroptotic cells; inhibition of PRDX3 in BPH-1 cells resulted in a decrease of LDH released into the culture medium. Therefore, PRDX3 in BPH may promote pyroptosis. While the available evidence suggests that the role of pyroptosis in BPH may not be obvious, maintaining a balance of proliferating and dying cells in the prostate by regulating pyroptosis may provide a new approach for BPH therapy.

## 4. Conclusion and perspectives

Cell death under physiological conditions (e.g., proper autophagy and apoptosis) is necessary to maintain tissue renewal, whereas under pathological conditions (e.g., autophagy, necroptosis, and pyroptosis) predispose to disease development and progression. Prostate diseases include PCa, prostatitis and BPH, all of which are serious threats to men’s health (85–87). A key feature of all three prostate diseases is the fact that they are all affected by inflammation. Downstream of pyroptosis is the inflammatory cascade response. Therefore, elucidating the role played by pyroptosis in different prostate diseases, which help to deepen our understanding of different prostate diseases.

It is the versatile roles of pyroptosis that dictate a variety of drug designs for different prostate diseases. Considering that pyroptosis is a double-edged sword in PCa, it is more complicated in designing drugs. Only by pinpointing the cancer cells in the prostate and prompting their pyroptosis can the therapeutic effect be better achieved. Therefore, there is an urgent need to address the problem of drugs targeting cancer cells in the prostate to induce pyroptosis. Nanoparticulate drug delivery systems (Nano-DDSs) have the potential to solve this problem with more targeted accumulation, slow release, the sustained onset of action, increased efficacy, and reduced toxicity than traditional drugs (88, 89). Hu et al. designed As2O3 nanoparticles for the treatment of hepatocellular carcinoma, which increased the expression of GSDME-N, promoted greater LDH release, and induced cancer cell pyroptosis compared to the free drug (90). Wand and co-workers via using a bioorthogonal chemical system found that desilylation catalyzed by phenylalanine trifluoroborate (Phe-BF3) could release an active GSDMD from a nanoparticle conjugate, which selectively into tumor cells in mice, thereby mediating pyroptosis to act against tumor cells (91). In the future, we can take full advantage of some biomaterials and combine them with the molecular mechanism of pyroptosis to design more targeted and lethal drugs for PCa therapy.

In prostatitis, anti-inflammation is one of the most influential forms of treatment (92). It is known that pyroptosis has a pro-inflammatory effect and anti-pyroptosis treatment in prostatitis may block the inflammatory response. Many Chinese herbs have shown beneficial anti-inflammatory effects in the treatment of prostatitis (93–95), but their mechanisms of action are not well understood. Therefore, whether they have an anti-pyroptosis function deserves further study. For BPH, a balance between cell proliferation and death is required. Therefore, it is difficult to design a treatment plan by pyroptosis. It is necessary to understand the role of pyroptosis in prostate disease.

In summary, the more comprehensive the understanding of the mechanisms of pyroptosis in prostate disease, the more accurate the diagnosis and prognosis of prostate disease. Moreover, a more comprehensive understanding of pyroptosis could provide more targeted and effective intervention strategies for prostate disease.

## Author contributions

MZ: conceptualization, formal analysis, writing—original draft, and review and editing. JG: conceptualization and supervision. Q-HG: writing—review and editing and formal analysis. HW, FW, Z-RW, Z-WZ, and Y-YZ: writing—review and editing. S-JL: data curation and formal analysis. Y-JD: data curation. W-XY: conceptualization, supervision, and writing—review and editing. All authors contributed to the article and approved the submitted version.
